# Exploring Quality of Life in Adults Living With Late-onset Pompe Disease: A Combined Quantitative and Qualitative Analysis of Patient Perceptions from Australia, France, Italy, and the Netherlands

**DOI:** 10.36469/001c.126018

**Published:** 2025-01-02

**Authors:** Holly Lumgair, Lisa Bashorum, Alasdair MacCulloch, Elizabeth Minas, George Timmins, Drago Bratkovic, Richard Perry, Medi Stone, Vasileios Blazos, Elisabetta Conti, Raymond Saich

**Affiliations:** 1 Amicus Therapeutics (United Kingdom); 2 Consultant for Amicus Therapeutics, Inc; 3 Women’s and Children’s Hospital, Adelaide, University of Adelaide; 4 Adelphi Values PROVE; 5 AiGlico, Assago, Italy; 6 Australian Pompe Association, Sydney, Australia

**Keywords:** late-onset Pompe disease, quality of life, patient perception, physical functioning, emotional wellbeing, patient-reported outcomes

## Abstract

**Background:** Late-onset Pompe disease (LOPD) is a rare, autosomal recessive metabolic disorder that is heterogeneous in disease presentation and progression. People with LOPD report a significantly lower physical, psychological, and social quality of life (QoL) than the general population. **Objectives:** This study investigated how individuals’ self-reported LOPD status (improving, stable, declining) relates to their QoL. Participant experiences such as use of mobility or ventilation aids, caregivers, symptomology, and daily life impacts were also characterized. **Methods:** A 2-part observational study was conducted online between October and December 2023 using the 36-item short-form tool (SF-36) and a survey. Adults with LOPD (N=41) from Australia, France, Italy, and the Netherlands were recruited. **Results:** Participants reporting “declining” LOPD status (56%) had lower physical functioning SF-36 scores than those reporting as “stable” or “improving.” Those self-reporting as stable or improving often described an acceptance of declining health in their responses. Physical functioning scores were generally stable in respondents who had been receiving enzyme replacement therapy (ERT) for 1-15 years, but those who had received ERT for >15 years had lower scores. Requiring ventilation and mobility aids had additive negative impacts on physical functioning. Difficulty swallowing, speaking, and scoliosis were the most burdensome symptoms reported by those on ERT for >15–25 years. **Discussion:** These results demonstrate the humanistic burden of LOPD; through declining physical functioning SF-36 scores over increasing time and increased use of aids, and also through factors related to self-reported LOPD status (where declining status was associated with lower scores) and symptomology variances. Taken holistically, these areas are valuable to explore when informing optimized care. Among a largely declining cohort, even those not self-reporting decline often assumed future deterioration, highlighting the need for improved therapies and the potential to initiate or switch ERT based on evolving symptomology and daily life impacts. **Conclusion:** Our results indicate that progressing LOPD leads to loss of QoL in ways that relate to time, use of aids, evolving symptomology, and the patient’s own perspective. A holistic approach to assessing the individual can help ensure relevant factors are investigated and held in balance, supporting optimized care.

## INTRODUCTION

Pompe disease is a rare, autosomal recessive metabolic disorder with an estimated prevalence that varies based on country and screening tools, but has been reported as ranging between approximately 1 per 4447 to 1 per 37 094 cases.^1^ It is caused by pathogenic variants in the acid α-glucosidase (GAA) gene with subsequent complete or partial impairment of endogenous GAA activity and impaired breakdown of lysosomal glycogen in body tissues.^2–4^ Phenotypically, Pompe disease can be broadly categorized into 2 clinical subtypes: infantile-onset (affecting infants <1 year of age) and late-onset Pompe disease (LOPD) (affecting both children and adults).^3^ The latter, which is the focus of this research, typically results in axial and limb-girdle patterns of skeletal and respiratory muscle weakness, as a consequence of lysosomal glycogen accumulation.^2,5^ As the disease progresses, patients commonly become wheelchair- and/or ventilator-dependent.^6,7^

Delays to diagnosis of LOPD are common due to the heterogeneity of disease presentation and progression.^8,9^ People with LOPD report a significantly lower physical, psychological, and social quality of life (QoL) than the general population.^10^ The impact of LOPD also extends to caregivers.^11^ As the disease progresses, LOPD often leads to respiratory failure, causing significant morbidity and mortality.^12–14^ In a published cohort study of untreated adults with LOPD, the median age at death was 55 years, and the observed mortality rate was higher than that of the general population.^14^

Currently the only approved treatments addressing the underlying cause of LOPD are enzyme-replacement therapies (ERTs), which involve biweekly infusions to augment the faulty GAA enzyme.^11,15,16^ The first ERT for LOPD, alglucosidase alfa (Myozyme^®^) received authorization by the European Medicines Agency in 2006.^17^ Although treatment with alglucosidase alfa has demonstrated improvements in respiratory and motor function in patients with LOPD, these changes are often not sustained in the long term and patients may experience secondary decline after 3 to 10 years of treatment.^18–20^ Thus, there is a need for LOPD treatments with improved effectiveness. Avalglucosidase alfa (Nexviadyme^®^) and cipaglucosidase alfa plus miglustat (Pombiliti^®^ plus Opfolda^®^) were authorized by the European Medicines Agency in 2022 and 2023 respectively, providing alternative ERT options for patients.^21–23^

Several studies have investigated the humanistic burden of LOPD, including the impact on QoL, the short-term and long-term effectiveness of ERT, and the associated day-to-day limitations.^10,11,18,24–26^ However, as LOPD is experienced differently by each individual, there is a lack of consensus on what constitutes meaningful improvement, stabilization, and decline from the perspective of patients.^27–29^ In this study, we sought to bring an additional dimension to understanding of QoL by deploying the 36-item short-form survey (SF-36) in combination with a multimodal survey, to understand how people’s perceptions of their self-described LOPD status (improving, stable, declining) related to their overall QoL. In addition, broader aspects such as use of mobility or ventilation aids, reliance on caregivers, individualized symptomology, and impact on daily life were captured in the participants’ own words, including the aspects of their lives that were impacted the greatest, to provide additional real-world context to the SF-36 findings.

## METHODS

### Study Design

This 2-part observational research was conducted online using the SF-36 in conjunction with a survey. The survey was developed by researchers with extensive experience in LOPD and was reviewed by both Pompe community members and patient advocacy organizations in participating countries, to ensure appropriateness and relevance.

### Inclusion Criteria and Recruitment

We sought to collect data from a total of approximately 40 adults with LOPD from Australia, France, Italy, and the Netherlands, aiming for an even distribution among the countries. Countries were chosen based on several criteria, including the size of the treated LOPD population, and with the support of the relevant local patient advocacy organizations, who helped with participant recruitment and survey launches. Participants were eligible for inclusion in this study if they met the following criteria: (1) were aged 18 years or older, had a confirmed LOPD diagnosis, (2) were receiving ERT at the time of the survey (outside the clinical trial setting), and (3) resided in 1 of the 4 countries. Prior to inclusion in the study, all participants were required to provide informed consent. Participant identities remained anonymous to the team conducting the study at all stages, and the survey did not request any personally identifiable information. All answers provided were kept confidential throughout the analytic process. The survey complied with all national laws protecting participant personal data and with relevant guidelines including European Pharmaceutical Market Research Association, the European Society for Opinion and Marketing Research, and Australian Market and Social Research Society. The study was reviewed and met the criteria for an exemption from an independent review board (Salus IRB).

### Data Collection

The study was conducted online between October and December 2023. It collected data on the QoL of people with LOPD and explored a range of variables, including their self-described LOPD status (as “stable,” “improving,” or “declining”) and self-described health status (as “poor,” “fair,” “good,” and “very good”), time on ERT, the frequency/severity of symptoms, and use of mobility aids and ventilation. The study comprised 2 parts: the SF-36 and a survey with qualitative and quantitative questions, asked at a single point in time. The SF-36 was selected as an appropriate validated tool for evaluating patient QoL in concordance with previously published LOPD literature.^12,30–33^ The survey used questions in multiple-choice and open-ended, verbatim formats, aiming to expand and provide further context around the SF-36 findings (**Supplemental Table S1**). Rating questions explored participant limitations due to their LOPD using a 9-point Likert scale, where 1 denoted “strongly disagree” and 9 denoted “strongly agree.” Where applicable, participants were encouraged to provide as much information as possible in their open-ended responses.

### Data Analysis

Following data collection, participant responses were collated and analyzed to identify key trends of the impact of different factors on participants’ self-described LOPD experiences. SF-36 scores were calculated based on the RAND scoring rules (Version 1.0).^34^ Findings were then stratified in relation to length of time on ERT, self-described LOPD status, self-described health status, use of mobility aids and/or ventilation (Yes/No) and the types/duration/frequency of aids used. Where necessary, responses were grouped; for instance, participants were categorized as ambulatory or nonambulatory, depending on their reliance on wheelchair assistance for mobility. Symptom frequency was analyzed in relation to SF-36 general health, physical functioning, and emotional well-being scores. The Pearson correlation coefficient was calculated to evaluate the strength and direction of the linear relationship between the SF-36 scores and 6-minute walking distance (6MWT)/forced vital capacity (FVC) results and statistical differences in SF-36 scores were analyzed at the 95% level (*P*<.05). The 6MWT and FVC are measures routinely used by clinicians to monitor LOPD progression.

### Qualitative Thematic Analysis

To further investigate patient perceptions of LOPD burden and impacts on daily life, a qualitative thematic analysis was conducted on participant free-text responses to relevant questions and compared between those self-described as declining or those self-described as stable or improving. As a small number of patients self-reported as improving, their responses were combined with patients self-reporting as stable. We used a rapid thematic analysis to analyze the free-text response data,^35,36^ which aimed to identify emerging patterns and themes within the data. The codebook was reviewed and edited for consensus among a primary coder and 2 reviewers. The codebook was designed to look for patterns between participants but also to support comparison across groups of respondents. All qualitative responses were coded using the codebook and then reviewed by the 2 reviewers. Upon confirmation of coded excerpts, the 2 reviewers and coder discussed the emerging findings among declining and stable or improving participants within and between the 2 groups.

## RESULTS

### Quantitative and Qualitative Analysis

Overall, a total of 41 people responded to the survey: 11 from Australia, 11 from France, 10 from the Netherlands, and 9 from Italy (**Supplemental Table S2**). When describing their health status as part of the SF-36, 9 (22%) participants reported poor, 11 (27%) reported fair, 16 (39%) reported good, and 5 (12%) reported very good health; no one reported excellent health. In terms of self-described LOPD status, 23 (56%) participants reported declining, 14 (34%) reported stable, and 4 (10%) reported improving health.

When declining participants were asked why this specific self-reported LOPD status had been selected, participants approached this question in a variety of ways. Key themes included describing decline in reference to specific physical symptoms such as pain and fatigue, as well as through ability to complete specific daily activities. Several considered how their current symptoms compared to prior levels of activity or pain, such as, “I’ve been noticing some worsening for a few months. I get more tired, I have some pain that I didn’t have before, I lost weight due to lack of appetite.” Participants described this in relation to the length of ERT use to refer to their disease experience. For example, “After 21 years, the effect of ERT is nil. The progression of the disease is in full swing again…” Among the 4 who selected an improving LOPD status, 3 (75%) described improvements in their mobility, breathing, and general physical abilities, and 1 (25%) described generally feeling better since beginning ERT. Participants who self-described their LOPD as stable or improving often utilized comparisons to their own internal benchmarks of progression, for example, “Before ERT, I would get cramping in my arms and my legs… After a year on ERT, they happen less, if at all.” Additionally, some used test results, such as, “On my first 6MWT I got 480 meters, then 6 months later, I got 680 meters so that was a very big improvement…” Important findings among the 18 participants who described their condition as stable or improving were that only one actually stated “I feel better!” and several used phrases demonstrating continued forms of deterioration including, “I feel like I’m getting less worse than when I didn’t do any therapy.”

Many participants across LOPD statuses directly referenced treatment as part of their assessment of their status. For example, “I perceive that ERT slows the progression of the pathology, but it is not sufficient to stabilize it in my case” and “Despite having taken ERT regularly without side effects for years, I completely lost the ability to walk and had to start using NIV [non-invasive ventilation] to sleep.”

When asked about the impact of their chosen self-described LOPD status on their daily lives, themes emerged from those declining related to impact on connection with family, ability to be social, and needing more help with daily activities. Among those stable or improving, some mentioned a positive impact on ability to work and autonomy, for example, “This allows me to still be autonomous on some tasks and maintain my professional activity.” However, participants noted an acceptance and assumption of decline even among those reporting improvement or stability, such as, “I have accepted that Pompe is a disease that is degenerative and I will slowly deteriorate,” and, “A little respite doesn’t seem bad to me; however, I remain very cautious for the future.” A clear demonstration of this understanding from one stable participant was, “Any deterioration is gradual, so you slowly get used to a limitation.”

Mobility limitations were a substantial factor in participants’ day-to-day lives. Some participants noted that they “can’t be autonomous,” they “were able to do less and less,” and that this had the biggest impact as it did not allow them “to live the life [they] want and do the activities [they would] like to do.” In the total cohort, the mean self-reported score for daily limitation of mobility was 7.4 (where 0 and 9 indicated the lowest and highest degrees of limitation, respectively). Among the 28 (68%) participants who reported that they required mobility aids as a result of their LOPD, the mean mobility limitation score was 8.5, while among the 13 (32%) who did not require mobility aids, the mean score associated with mobility limitation was 5.2. Mobility aids included walking sticks, rails, and wheelchairs, and 13 (32%) participants required more than 1 physical aid.

In terms of breathing, the mean score associated with daily limitation was 5.5 (where 0 and 9 indicated the lowest and highest degrees of limitation, respectively). Among the 18 (44%) participants who reported that they required ventilation aids, the mean score associated with breathing limitations was 6.8, while the 23 (56%) who did not require ventilation aids reported a mean score of 4.5.

Approximately half (46%) of the participants in the study reported that they were able to function independently of any other assistance; however, 22 (54%) participants required a caregiver. Participants believed that their condition had a substantial impact on their non-professional caregivers, reporting a mean score of 7.3 for their LOPD limiting their everyday lives (where 0 and 9 indicated the lowest and highest degrees of limitation, respectively). Caregivers assisted with daily activities, such as cleaning, cooking, or taking care of children.

Thirty-two (78%) participants reported they had previously completed the 6MWT, with the primary reason for a 6MWT not being undertaken being due to inability to walk independently. The mean 6MWT among the 20 (49%) participants who knew their most recent score was 429.1 meters. Similarly, the majority had had their FVC assessed in the past (85%); reasons for never having an FVC measurement were not knowing what it was, its importance, or never being offered to complete it during healthcare checkups. The mean FVC in the 11 (27%) participants who knew their most recent score was 57.5% of the predicted value.

### SF-36 Scores and Trends

**Figure 1.**
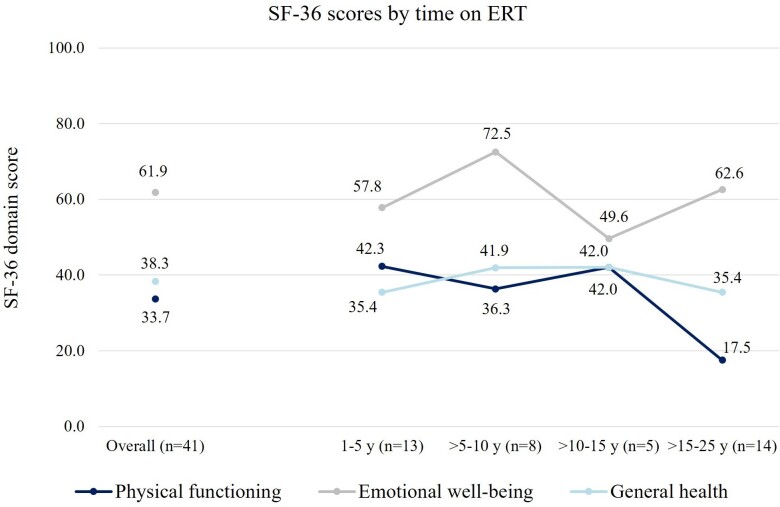
SF-36 Scores by Time on Enzyme Replacement Therapy Abbreviations: ERT, enzyme-replacement therapy; SF-36, 36-item short-form survey.

**Figure 2.**
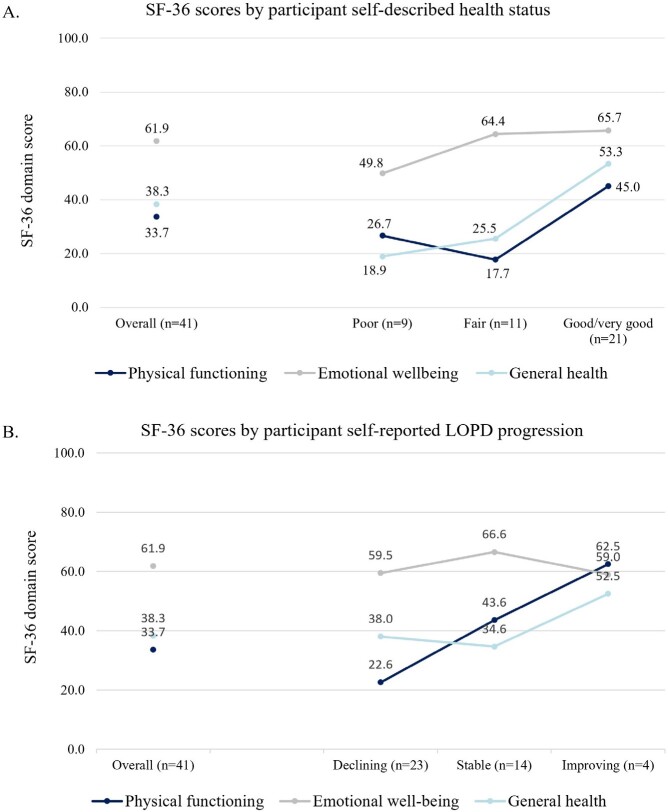
SF-36 Scores by (**A**) Self-Described Health Status and (**B**) Self-Reported LOPD Status Abbreviations: LOPD, late-onset Pompe disease; SF-36, 36-item short-form survey.

The mean (SD) general health score among participants was 38.3 (21.1), where 0 indicates the worst and 100 indicates the best possible condition. Mean (SD) physical functioning was significantly lower than mean emotional well-being, with reported scores of 33.7 (29.1) and 61.9 (23.1), respectively (*P* < .05). Physical functioning scores were substantially lower among participants that were on ERT for longer, while general health or emotional well-being scores were comparable between individuals with different lengths on ERT (**Figure 1**). In this survey, physical functioning was relatively stable in individuals who had received ERT for ≤15 years, but with lower scores presenting in those with >15 to 25 years on ERT. Notably, 2 people in this survey had been on ERT for >20 to 25 years, and both reported physical functioning scores of zero. In general, SF-36 scores aligned with participants’ perceptions of their condition, increasing as reported health status changed from poor, to fair, to good/very good (**Figure 2A**). Physical functioning scores were substantially higher for participants whose health was reported as improving, whereas emotional well-being scores were generally stable among those who reported their condition as improving/stable/declining (**Figure 2B**).

**Figure 3.**
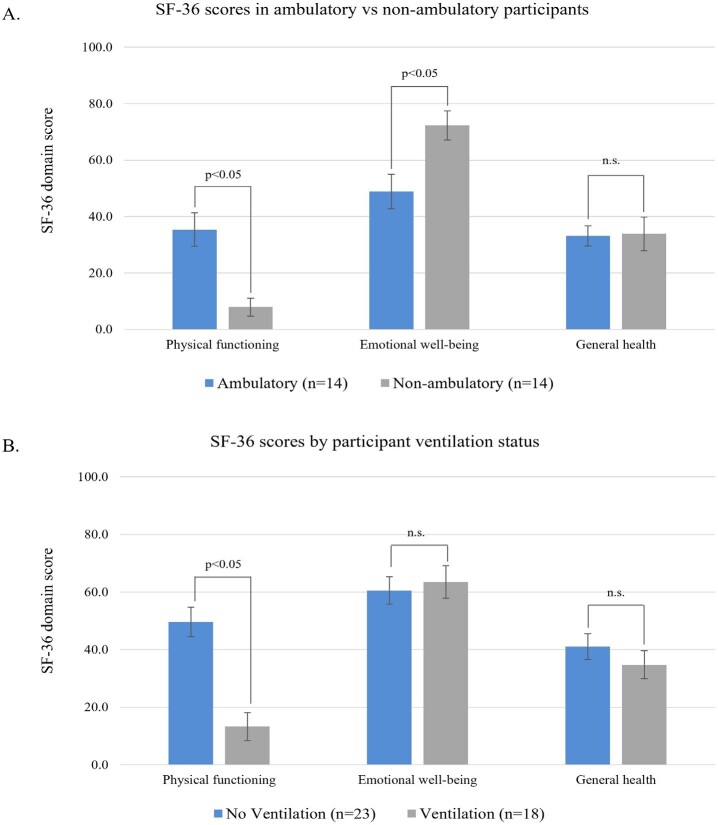
SF-36 Scores by (**A**) Mobility Assistance Status (Ambulatory vs Nonambulatory) and (**B**) Ventilation Status Abbreviation: SF-36, 36-item short-form survey.

**Figure 4.**
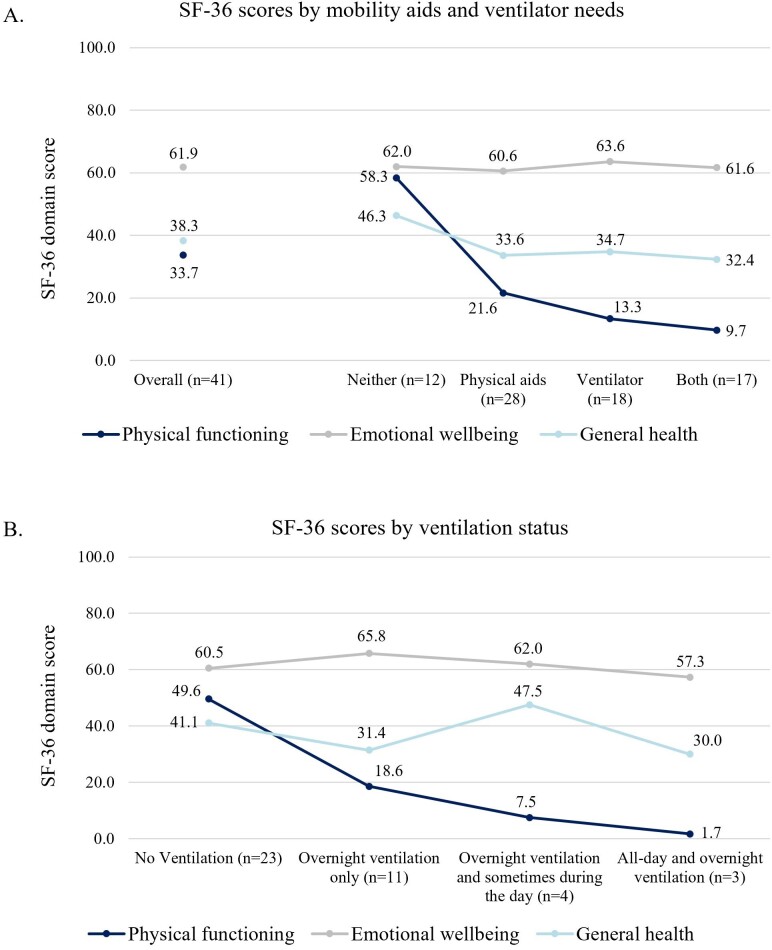
SF-36 Scores by (**A**) Use of Mobility and/or Ventilation Aids and (**B**) Ventilation Frequency Abbreviation: SF-36, 36-item short-form survey.

When stratifying SF-36 scores by physical aid and ventilation requirements, physical components were impacted substantially more than emotional well-being (**Figure 3**). Ambulatory participants had significantly higher physical functioning but significantly lower emotional well-being scores than nonambulatory participants. Participants on ventilation reported significantly lower physical functioning than those not on ventilation (*P* < .05), although there were no significant differences in emotional well-being between the 2 groups. Being on ventilation and requiring mobility aids had an additive negative impact on physical functioning scores, although emotional well-being remained relatively stable regardless of use of mobility and/or ventilation aids (**Figure 4A**). Physical functioning was substantially lower in people using all-day and night ventilation, followed by those using ventilation overnight and sometimes during the day, and those only requiring ventilation overnight. Physical functioning was substantially higher in participants that did not use ventilation, although emotional well-being and general health were relatively similar to those with ventilation requirements (**Figure 4B**). Physical functioning scores were strongly positively correlated with both 6MWT and FVC scores (Pearson correlation coefficient, *r* = 0.80 and *r* = 0.66, respectively) (**Supplemental Figure S1**).

Emotional well-being was significantly higher than general health and physical functioning and remained similar irrespective of participant self-reported LOPD status. Scores also remained relatively stable irrespective of duration of ERT. This is unlike physical functioning scores, which were substantially lower among those that were on ERT for longer periods. Physical functioning was highest 1 to 5 years post-ERT initiation; however this declined as time on ERT increased beyond 10 to 15 years.

### Symptoms

**Figure 5.**
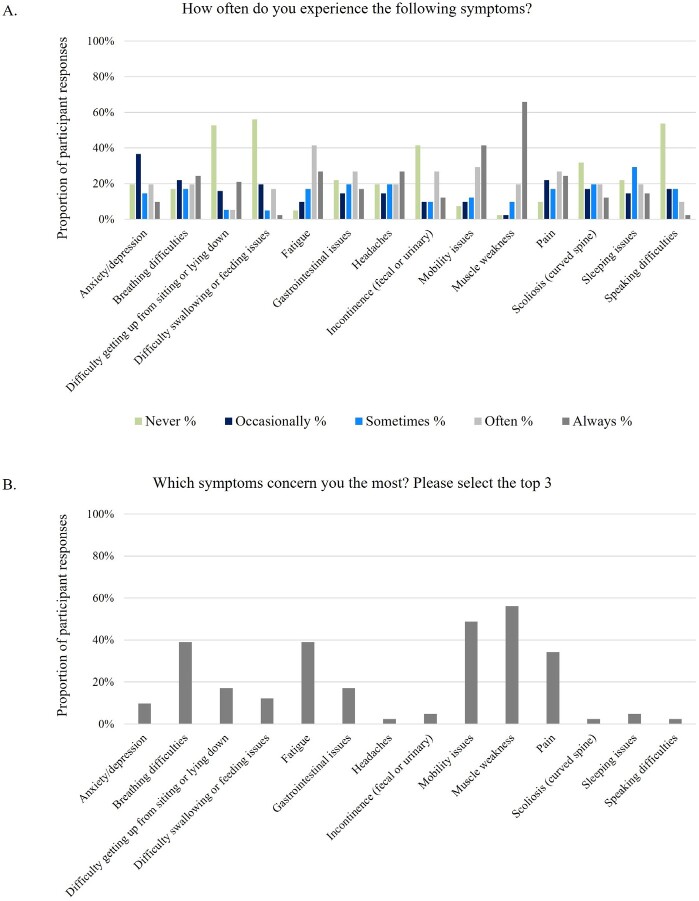
Most Frequent (A) and Most Concerning or Burdensome (B) Self-Reported Symptoms of People With LOPD Abbreviation: LOPD, late-onset Pompe disease. Specific scoliosis symptoms were not explicitly explored.

The most frequently reported symptoms were also reported as being the most concerning or burdensome (**Figure 5**). The 5 most concerning symptoms were muscle weakness, fatigue, mobility issues, pain, and breathing difficulties, which were experienced by 40 (98%), 39 (95%), 38 (93%), 37 (90%), and 34 (83%) participants, respectively. These symptoms were experienced consistently by participants regardless of the duration they had been receiving ERT, while others, (eg, scoliosis, sleeping issues, and speaking difficulties) were more frequently reported among those that had been on ERT for longer (**Supplemental Figure S2**). Difficulty swallowing was only reported among the most concerning or burdensome symptoms by 5 (12%) participants, who had been on ERT for >15 to 25 years, while scoliosis and speaking difficulties were only reported among the most concerning or burdensome symptoms once each (2%) by 2 participants, both who were on ERT for >15 to 25 years.

When categorizing responses by SF-36 domain scores, some symptoms were more frequently reported among those with lower scores while others were consistently reported throughout the cohort. Participants with lower physical functioning scores noted a higher frequency of mobility issues, gastrointestinal issues, incontinence, difficulty swallowing, scoliosis, and speaking difficulties, while fatigue, muscle weakness, pain, and breathing issues were reported at similar levels throughout. Among participants with lower emotional well-being scores, difficulty swallowing, speaking difficulties, anxiety, incontinence, headaches, and breathing issues were reported more frequently than other symptoms. Difficulty getting up from sitting/lying down was the only symptom that was more frequently reported in those with higher emotional well-being scores.

### LOPD Impact on Day-to-Day Life

Participants reported that LOPD substantially limited their daily life (**Table 1**). Work was the most impacted aspect of daily life (n = 30 [73%] reporting that LOPD had a high impact on their work life), followed by ability to travel (n = 24 [59%]), social life (n = 23 [56%]), family life (n = 19 [46%]), and then education (n = 4 [10%]). Participants noted a “huge” impact on their work, reporting feeling “stuck and worthless,” with 22 (55%) having to reduce their workload or stop working entirely. Regarding travel, participants most noted that they had to reduce the frequency and duration of their trips, as well as requiring “careful planning” due to pain and the risk of hospitalization being considerably limiting. Participants noted similar feelings about their social and family lives, reporting that their relationships “fell apart,” feelings of “isolation,” as well as being too “tired” to “go out.” The majority noted that they were adults and therefore not in education anymore. Among the 4 (10%) participants who reported a high impact of LOPD on their education, 2 noted that it limited their ability to attend school, while the other 2 noted feeling “stigmatized” and being the target of “mockery” because of their condition. A substantial proportion (n = 28 [68%]) also noted that LOPD limited their future plans, characterized by demotivation and negative feelings about the progression of their condition. Some participants noted that they were unable to plan for their future, “being afraid” of their condition getting worse and being uncertain about how it would progress over time.

**Table 1.** Qualitative Survey Responses to the Impact of LOPD on Domains of Daily Life

**QoL Theme**	**No. (%) of Participants**	**Key Qualitative Statements**
Low impact		
Work	4 (10)	“I think my work has really helped my physical ability every day I push myself maybe that’s why I’m so tired, but I think it has stopped me from being as bad.” “Doesn’t majorly affect my job fortunately.” “[I] just work to earn income. LOPD does not absolve me of my duty to take good care of myself.”
Education	20 (49)	“I don’t think it has ever affected my education every day I learn new lessons.” “Not much [impact].” “It’s not an obstacle.”
Travel	5 (12)	“I live very close to work so either drive or use my motorized mobility devices.” “Just don’t postpone anything, do what you can do now. I have been to many countries (India, China, Australia, USA, Canada, Turkey, Syria, Lebanon, Jordan, and Europe).” “It has not had any influence on my way of traveling so far”
Social life	8 (20)	“It doesn’t affect my social life I still play sport (lawn bowls) 3-4 times a week and still all the normal things I did before I was diagnosed”. “No real big impact, I continue my social activities.” “I still see friends and family.”
Family life	8 (20)	“I’m just starting a family with a baby due […] and just got married […] so I’ll just have to see, but hopefully it shouldn’t affect it too much.” “Family is very present, but that brings us even closer.” “Not too limiting.” “This is still going quite well, but everyone in the house helps. Like lifting the laundry basket upstairs for me.”
Future plans	5 (12)	“I hope it won’t affect any future plans. Everyone is different with Pompe for myself, I feel pretty stable right now and happy with how my life is living with Pompe and we just have to see what the future brings.” “To be seen over time.” “No [impact] as there is a stabilization and I limit my plans.” “I can worry sometimes, but I still see a bright future ahead of me. Whether or not in a wheelchair…”
Moderate impact		
Work	2 (5)	“It doesn’t stop me from doing my job. I’m just a little slower than everyone else and need help with lifting heavy things around [the] site. All my work mates are happy to help in these areas.” “Limits me in [work] activities.”
Education	4 (10)	“It depends: if the institutions guarantee access for disabled people its impact is limited, if this does not happen it prevents me from taking university or training courses.” “I’m still a student. And because of my ERT I sometimes miss classes. This means that I have to actively monitor my schedule and try to catch up on lessons I miss by doing self-study or following a different schedule.”
Travel	11 (27)	“I can still drive. Getting public transport when needed is fine too. Unable to access certain places that have stairs or rough and or uneven terrain.” “Not being able to take certain organized trips, check that the sites are accessible, no longer go hiking. On the other hand, for means of transport such as train or plane, the support is well done. This is not true for transport such as the metro or buses.” “I need a companion.”
Social life	8 (20)	“I can’t go to certain events if they aren’t accessible, and I do sometimes feel like a bit of a burden when I have to rely on friends. It does also limit activities I can do with my friends and family.” “A relative limitation, I cannot participate when there are sporting events.” “Because of my ERT I sometimes have to cancel spontaneous appointments that are made.”
Family life	12 (29)	“I cannot be there to help them as much as I would like. I try to stay at home so family can still go do things they enjoy.” “I can sometimes be irritable which sometimes makes relationships with my partner complicated.” “It limits me in managing the house.” “Everyone takes my ERT into account to some extent, for example when on holiday.”
Future plans	6 (15)	“I live one day at a time; I had to leave my house and readjust a new place to be ready when things no longer work out.” “I hope not much.”
High impact		
Work	30 (73)	“I had to leave my career.” “Huge [impact]. I feel stuck and worthless.” “I was forced to gradually reduce my working time until I was permanently incapacitated following the placement on category 2 disability. This was very complicated to accept because we once again feel excluded from society, and we suffer illness.” “I started working less and in a lower position.”
Education	4 (10)	“The high school period was complicated because [my] symptoms were considered and research began, leading to a lot of hospitalizations, appointments, and medical examinations… my fatigue was a source of mockery when there was no diagnosis yet.” “I was stigmatized because people thought I was lazy or that I had bad will. Teachers and classmates didn’t understand why I wasn’t present for certain classes (infusion days) even though I had informed them of my treatment. I was stigmatized.” “Massively, sometimes I am unable to attend.” “I did not follow the training that I would otherwise have taken without LOPD.”
Travel	24 (59)	“On long drives it hurts and takes me about 2 minutes to walk from siting down for so long”. “I limit my travels. I prevent myself from traveling to certain countries for fear that I will have to be hospitalized or have digestive problems or poorer health conditions... I also prevent myself from going on organized trips because if I am unsure about the excursion times, in the morning I need time.” “I don’t travel by plane for fear of illness and how the aids are treated. I travel by car only if essential (for medical visits).” “I can no longer walk or walk much, so it does hinder me from going out for days.”
Social life	23 (56)	“I don’t have one apart from computer games with mates.” “I don’t go out anymore, I’m too tired.” “Huge. I am not satisfied with my ability to socialize because despite my sociable character. I am limited in everything.” “Big [impact]. [I have a] limited social life due to fatigue, among other things.”
Family life	19 (46)	“It limits activities and there is no room for spontaneity. Everything must be planned and ensure that it is accessible. I do sometimes feel a bit left out or that my partner does have to pick up the slack.” “Apart from my parents who understand me, for my partner and my children it remains complicated to understand.” “Huge [impact], not being able to detach myself from my family makes me suffer.” “I look after my grandchildren very little. Now that they are older it can be easier, but I miss not being able to do all kinds of things with them.” “My marriage fell apart after my diagnosis. I now live on my own with my dog and find that it is a lot easier if there is no one around. I don’t have to explain myself if I’m having a bad day.”
Future plans	28 (68)	“Very much, I expect my health to get worse, so it is very difficult for me to hope for a better future.” “I can’t plan for the long term because I don’t know how the disease will progress. I’m probably limiting myself wrongly. But I need to be afraid of the future.” “I don’t think I will have a future.” “… it is uncertain what the future will look like.”

## DISCUSSION

This study combined the SF-36 with qualitative statements from people with LOPD aiming to understand the heterogeneous nature of LOPD and the implications for improving standard care and future therapies. The mean SF-36 general health, physical, and emotional scores were 38.3, 33.7, and 61.9, respectively. These results highlight the substantial humanistic burden of LOPD, particularly in respect to general health and physical functioning. The SF-36 scores in our study align with previously published studies evaluating the QoL of people living with LOPD using the SF-36.^30,37^ General health, physical functioning, and emotional well-being scores for our cohort were lower than for the respective country general populations, as well as being lower than SF-36 scores reported in people with other rare genetic disorders such as hemophilia A and Fabry disease.^38–44^

When participants were asked to describe their current LOPD status, the majority stated that they were declining. This was based on their current symptomology, the impact on their daily life, or an internalized standard (eg, an activity of daily living becoming easier or more difficult to achieve). ERT use was often mentioned by patients as an anchor point against which disease status was measured. Further, participants who reported stable or improving condition still often described their health in terms of an acceptance and assumption of ongoing and future decline. Therefore, while current treatment is slowing disease progression and allowing temporary improvements in people’s daily lives, patients are still living with the assumption of their worsening condition, indicating a need for improved treatment options and wider support.

General health and physical functioning SF-36 scores were substantially lower among those reporting poor health status and declining LOPD status. This suggests self-reported LOPD status is associated with general health and physical functioning scores. People with LOPD should proactively inform healthcare professionals of their perceived health, even when there is no change in their standardized test results, as earlier intervention may help to maintain their physical functioning and general health. Furthermore, incorporating individual patient perspectives of LOPD status and its impact alongside clinical evaluation will allow for more holistic and collaborative care decisions to be made for people living with LOPD.

Other studies have also reported a decline in 6MWT and FVC after 2 to 5 years of treatment.^19,45^ These findings are similar to those from a single-center observational study in the United Kingdom, which reported that a transition to ERT improved mobility and breathing for up to 5 years post-ERT before declining, and that mental health remained unaffected throughout.^18^ Within our study, the majority of participants self-reporting declining LOPD status were on ERT for more than 10 years (60%) and of the 4 participants reporting improving health status, 1 had recently switched ERT and 2 recently initiated ERT for the first time. This highlights the need for newer therapies to alleviate the physical decline observed over time on ERT and the potential to initiate or switch ERT based on patients’ evolving symptomology. Further, the maintained emotional well-being suggests that people with LOPD tend to experience a cognitive adjustment to their reduced physical functioning.

Interestingly, participants requiring mobility assistance, ventilation, or both had higher emotional well-being scores than those without. It appeared that mobility aids such as wheelchairs, ramps, and walking sticks, and ventilatory support, may help to improve the QoL of people with LOPD. Physical scores among participants requiring both physical and respiratory aids were substantially lower than those using just one or the other, although emotional scores were omparable between all groups. Though the use of mobility aids and ventilation by people with LOPD has been explored in the past, this is the first published study to evaluate the additive impact of requiring both.^18,46^ The emotional benefit conferred by these aids may be due to people with LOPD placing higher value on minor improvements that enable them to carry out simple day-to-day activities, as suggested by some of the responses in our survey. Participants did note some negative emotions associated with being reliant on devices such as wheelchairs, ramps, and non-invasive ventilation; these are often bulky and inconvenient. However, they largely described that these aids allowed them to achieve a sense of independence and baseline functioning that would be unattainable otherwise.

Large proportions of participants in our study had previously completed the 6MWT (78%) and tested their FVC (85%). Due to heterogeneity in LOPD, clinical measurements, such as 6MWT and FVC, may not always be appropriate for patients (eg, those who are nonambulatory or those requiring invasive ventilatory support, respectively) and are of limited utility in isolation. It is imperative that patient LOPD status is measured holistically through other parameters, including clinical domains, patient-reported outcomes, as well as the individual’s own experienced impact on daily life. Healthcare professionals should explore alternative monitoring techniques on a case-by-case basis and consider direct patient input of their experiences (including symptomology), in conjunction with standard clinical assessments.

Previous publications have evaluated the relationship between improved functional test scores and patient-reported outcomes (PROs) in LOPD and suggest that mobility and respiratory improvements can help to improve QoL.^47,48^ A cross-sectional study of 121 Dutch people with LOPD reported that 6MWT and FVC functional test scores had a strong positive correlation with the physical, but not mental, component of the SF-36.^49^ These findings align with our study, further suggesting that changes in functional test scores in people with LOPD align with physical improvements captured by the SF-36; however, they do not result in improved emotional health outcomes.

To our knowledge, this is the first study exploring the frequency and severity of different LOPD symptoms, analyzed by time on ERT, SF-36 scores, and patients’ perception of their status (improving, stable, or declining). The most frequently reported symptoms in our cohort population were mobility issues, muscle weakness, and fatigue. This is similar to the findings of a recent observational study that reported walking difficulties and fatigue as the most frequent symptoms.^26^ Here, we identified that the most frequently reported symptoms were also among the most concerning or burdensome. Increasing use of aids that support with managing these symptoms may initially positively impact physical functioning scores within the SF-36. Pain and fatigue, in particular, were associated with declining LOPD status, and therefore healthcare providers should aim to manage these symptoms. Some symptoms, including mobility issues, muscle weakness, pain, fatigue, and breathing issues, were consistently self-reported to be among the most concerning or burdensome, while others were only reported in participants who have been on ERT for longer. For example, difficulty swallowing was only mentioned by participants on ERT >15 to 25 years, and scoliosis and speech difficulties were only noted among the most concerning or burdensome by 2 participants who both were on ERT for >15 to 25 years. The disproportionate burden of these symptoms highlights the progressive nature of LOPD and suggests that some symptoms are typically only experienced in people with LOPD further along their disease experience. This highlights the need for healthcare professionals to carefully monitor symptomology as new or worsening symptoms may indicate declining health.

Symptom frequency also varied by SF-36 scores, suggesting that certain symptoms posed greater limitations upon different QoL domains than others. Fatigue and pain were consistently reported throughout the cohort, whereas mobility issues, gastrointestinal issues, incontinence, swallowing difficulties, scoliosis, and speaking difficulties were reported more frequently by patients with lower physical functioning scores. Further, speaking or swallowing difficulties, anxiety, incontinence, headaches, and breathing issues were more frequent among participants with lower emotional well-being scores. Difficulty getting up from sitting or lying down was the only symptom reported more frequently by patients with higher emotional scores, as it is commonly one of the first symptoms that manifest during people’s LOPD experience. It is important that people with LOPD are encouraged to communicate the symptoms they find most burdensome to aid healthcare providers in understanding how to best approach their clinical management. In such cases, symptom-specific PROs may be preferable to generic tools to obtain a deeper understanding of the impact of symptoms as LOPD progresses.^11,50^ The 2024 European Pompe Consortium guidelines recommend the Fatigue Severity Scale and Brief Pain Inventory scale as key PROs for the assessment of two of the most burdensome symptoms in people with LOPD.^51^

The day-to-day limitations as a result of symptoms were explored on a quantitative basis, supplemented by patient statements to contextualize findings. Participants reported substantial limitations as a result of their LOPD, noting a high impact on their work (73%), travel (59%), social (56%), and family lives (19%), respectively. Education was the only aspect of life that was not significantly impacted, primarily due to the older population of the cohort, meaning that participants were not in education at the time of the survey. Participants reported a high impact on their future life plans (68% of the total cohort), which was reflected by the qualitative statements provided. Feelings of hopelessness, lack of ambition and pessimism were very common throughout. This has been demonstrated among people with rare diseases in previous literature, with studies showing that marginalization, bullying, victimization, and social isolation are common and lead to an altered sense of life aspiration.^52,53^

## CONCLUSION

People with LOPD experience substantial limitations that place a significant burden on their day-to-day lives. This is the first study combining SF-36, 6MWT, and FVC scores with a multimodal survey to compare the measurable QoL of people with LOPD and their individual perceptions of their disease status over several different variables. Participants in our cohort reported high levels (56%) of decline, which correlated with lower general health and physical functioning SF-36 scores and increasing ERT duration. Declining physical functioning was associated with increasing use of both physical and ventilatory aids; however, emotional well-being remained stable with time on ERT and with increasing use of aids. Also, patients self-reporting lower health or with a perception of a declining LOPD status scored lower on the SF-36 physical functioning domain. The symptomology of people with LOPD increased and worsened over time, with symptoms such as swallowing difficulties and scoliosis newly appearing in participants that had been receiving ERT for >15 years. This study underscores that clinical measurements such as the 6MWT or FVC in isolation are not always sufficient to assess ongoing disease progression and the impact on patients’ QoL, due to the heterogeneity of LOPD. Therefore, a holistic approach is necessary, one that considers individual symptomology, perspectives, and daily life impacts.

## Supplementary Material

Online Supplementary Material
