# Supplementary material for: Exploring Quality of Life in Adults Living With Late-onset Pompe Disease: A Combined Quantitative and Qualitative Analysis of Patient Perceptions from Australia, France, Italy, and the Netherlands
Source: J Health Econ Outcomes Res. 2025 Jan 2;12(1):1–12. doi: 10.36469/001c.126018 (PMC11699855; doi:10.36469/001c.126018)
Supplement: Online Supplementary Material [file jheor_2025_12_1_126018_260226.pdf]

Journal of Health Economics and Outcomes Research

Online Supplementary Material

Exploring Quality of Life in Adults Living With Late-Onset Pompe Disease: A Combined Quantitative and Qualitative Analysis of Patient Perceptions from Australia, France, Italy, and the Netherlands. *JHEOR*. 2025;12(1):1-12. doi:10.36469/jheor.2025.126018

Table S1: Key Themes Explored in the Survey

Table S2: Participant Characteristics

Figure S1: Association of Participant SF-36 Physical Functioning Scores and 6MWT/FVC Scores

Figure S2: Frequency of Self-Reported Symptoms Among the Most Concerning or Burdensome by Time on Enzyme Replacement Therapy

This supplementary material has been provided by the authors to give readers additional information about their work.

This is an open-access article distributed under the terms of the Creative Commons Attribution 4.0 International License (CCBY-4.0). View this license's legal deed at http://creativecommons.org/licenses/by/4.0 and legal code at http://creativecommons.org/licenses/by/4.0/legalcode for more information.

Table S1. Key Themes Explored in the Survey

Topic	Key Themes Explored
Disease status	Length of time on ERT<br>LOPD perceived symptom status<br>How this status affects day-to-day life<br>Suggested improvements on treatments and beyond
Symptoms and diagnosis	Age at which experienced symptoms for the first time<br>Time from first experiencing symptoms to diagnosis<br>Importance of what an earlier diagnosis would mean to participants
Physical functioning	6MWT and FVC scores, where available<br>Reasons why 6MWT or FVC tests were not completed<br>Mobility limitation impact on day-to-day life<br>Mobility aids and their impact<br>Breathing limitation impact on day-to-day life<br>Impact of ventilation<br>Caregiver support requirements
Burden of symptoms	Symptoms most commonly experienced<br>Most concerning or burdensome symptoms and their impact on day-to-day life<br>Progression status of symptoms and their impact on day-to-day life
Effect on day-to-day life and future plans	Impact of LOPD on work, education, travel, social and family life<br>Impact of LOPD on future plans

Abbreviations: 6MWT, 6-minute walking test; ERT, enzyme-replacement therapy; FVC, forced vital capacity; LOPD, late-onset Pompe disease.

Table S2. Participant Characteristics

Category	Value
Symptoms and treatment	
Age first experiences symptoms, mean (range)	17.4 years (range 1-2 years)
Delay between first experiencing symptoms and a diagnosis of LOPD, among participants diagnosed after displaying symptoms of LOPD, mean (range)	22.5 years (0-57 years).
Receiving treatment for another condition (eg, hypothyroidism, asthma, and hypertension), n (%)	15 (37%)
Time on ERT, mean (range)	9 years 9 months (9 months–21 years 3 months)
Mobility aids	
Do not require mobility aids, n (%)	13 (32)
Require mobility aids, n (%)	28 (68)
Require mobility aids and ambulatory[a], n (%)	14 (34)
Require mobility aids and nonambulatory[a], n (%)	14 (34)
Ventilation aids	
Do not require ventilation aids, n (%)	23 (66)
Require ventilation aids, n (%)	18 (44)
Overnight use of ventilation, n (%)	11 (27)
All-day and night use of ventilation, n (%)	3 (7)
Overnight use of ventilation and sometimes during the day, n (%)	4 (10)
VPAP, n (%)	14 (34)
BiPAP, n (%)	4 (10)
Caregivers	
Function independent of assistance, n (%)	19 (46)
Require a caregiver, n (%)	22 (54)
Time requirements for professional caregiver, mean	29 hours/week
Time requirements for family member/friend support, mean	37.3 hours/week

[a]Ambulatory people were defined based on their reliance on wheelchair assistance for mobility.
Abbreviations: BiPAP, biphasic positive airway pressure; VPAP, variable positive airway pressure.

Figure S1. Association of Participant SF-36 Physical Functioning Scores and 6MWT/FVC Scores

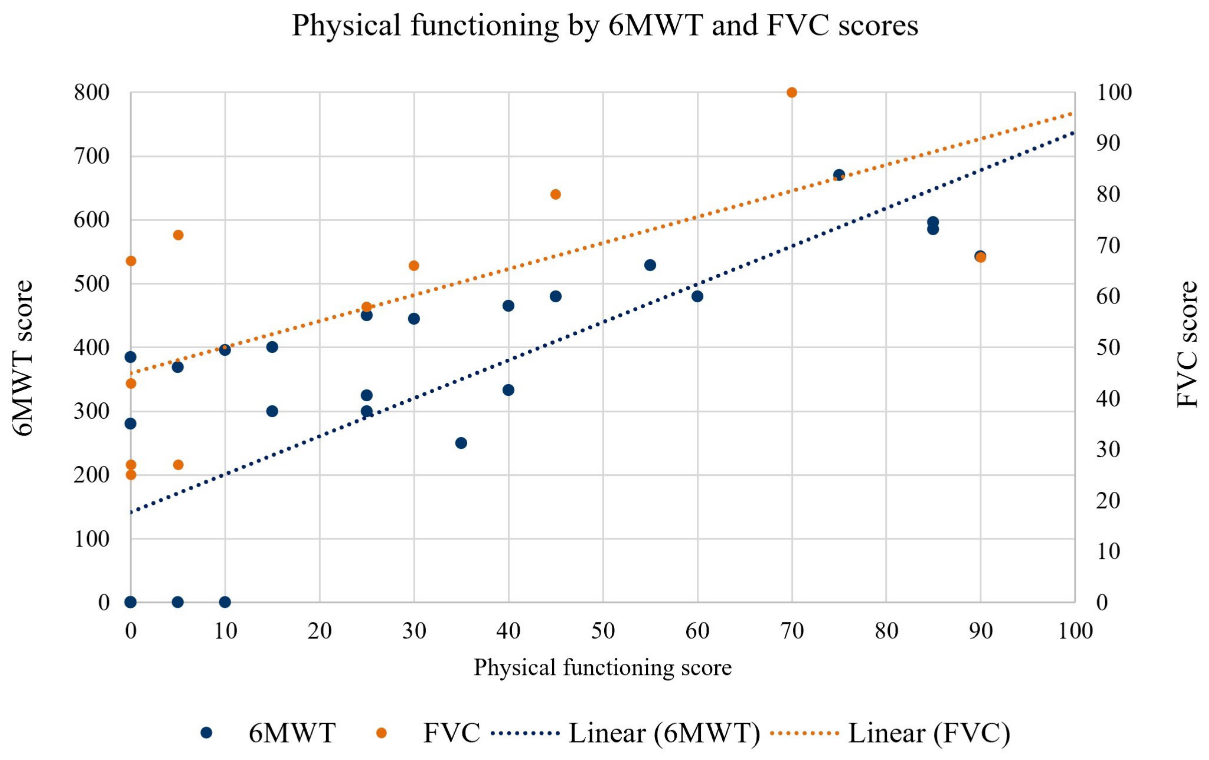

Abbreviations: 6MWT, 6-minute walking test; FVC, forced vital capacity; SF-36, 36-item short-form survey.

Figure S2. Frequency of Self-Reported Symptoms Among the Most Concerning or Burdensome by Time on Enzyme Replacement Therapy

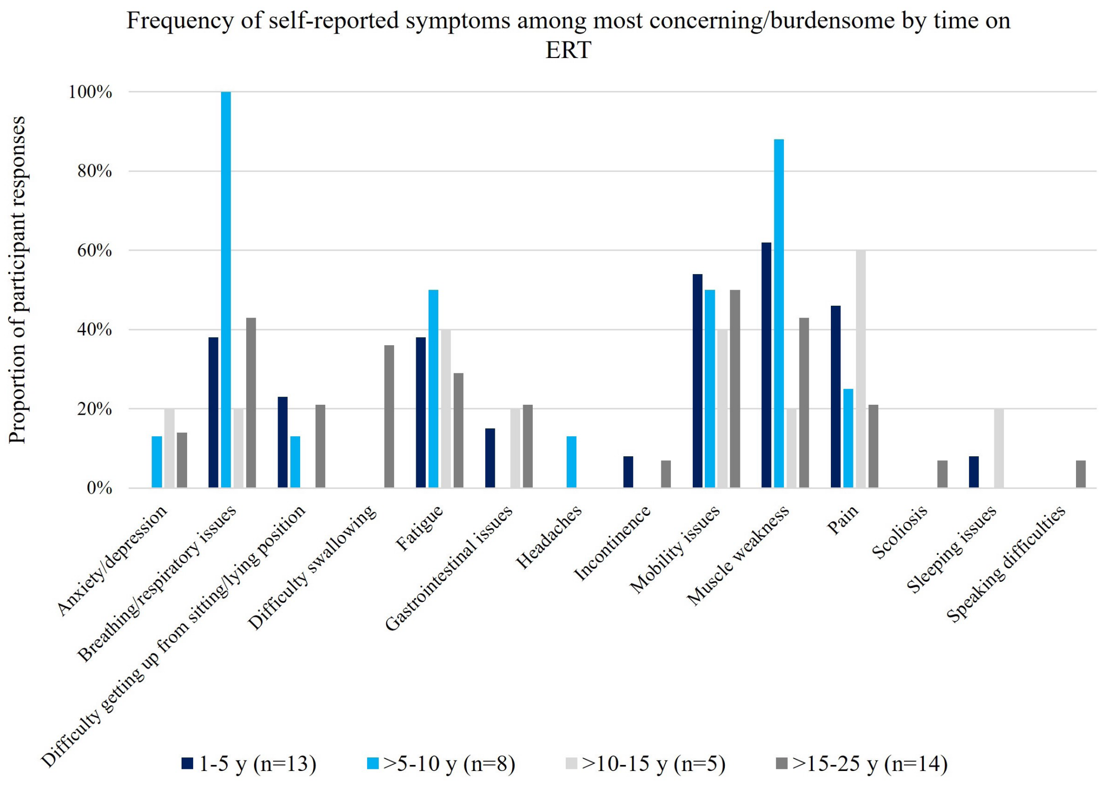

Abbreviation: ERT, enzyme-replacement therapy.
